# Influence of Non-Thermal Plasma Treatment on Structural Network Attributes of Wheat Flour and Respective Dough

**DOI:** 10.3390/foods12102056

**Published:** 2023-05-19

**Authors:** Muhammad Jehanzaib Khan, Vojislav Jovicic, Ana Zbogar-Rasic, Viktoria Zettel, Antonio Delgado, Bernd Hitzmann

**Affiliations:** 1 Institute of Fluid Mechanics (LSTM), Friedrich-Alexander-University Erlangen-Nuremberg (FAU), 91058 Erlangen, Germany; vojislav.jovicic@fau.de (V.J.); ana.zbogar-rasic@fau.de (A.Z.-R.); antonio.delgado@fau.de (A.D.); 2 Department of Process Analytics and Cereal Science, University of Hohenheim, 70599 Stuttgart, Germany; viktoria.zettel@uni-hohenheim.de (V.Z.); bernd.hitzmann@uni-hohenheim.de (B.H.); 3 German Engineering Research and Development Center, LSTME Busan, Busan 46742, Republic of Korea

**Keywords:** wheat flour, cold plasma, dough rheology, microstructure, protein and starch properties, hydration, microbial activity

## Abstract

Due to its “generally recognized as safe status” (GRAS) and moderate treatment temperatures, non-thermal plasma (NTP) has lately been considered a suitable replacement for chemicals in the modification of food properties and for preserving food quality. One of the promising areas for the application of NTP is the treatment of wheat flour, leading to improved flour properties and product quality and consequently to higher customer satisfaction. In the present research, the German wheat flour type 550, equivalent to all-purpose flour, was treated using NTP in a rotational reactor to determine the influence of short treatment times (≤5 min) on the properties of flour (moisture and fat content, protein, starch, color, microbial activity, and enzymes), dough (visco-elastic properties, starch, wet and dry gluten, and water absorption), and baking products (color, freshness, baked volume, crumb structure, softness, and elasticity). Based on the properties of NTP, it was expected that even very short treatment times would have a significant effect on the flour particles, which could positively affect the quality of the final baking product. Overall, the experimental analysis showed a positive effect of NTP treatment of wheat flour, e.g., decreased water activity value (<0.7), which is known to positively affect flour stability and product shelf life; dough stability increased (>8% after 5 min. treatment); dough extensibility increased (ca. 30% after 3 min treatment); etc. Regarding the baking product, further positive effects were detected, e.g., enhanced product volume (>9%), improved crumb whiteness/decreased crumb yellowness, softening of breadcrumb without a change in elasticity, and limited microorganism and enzymatic activity. Furthermore, no negative effects on the product quality were observed, even though further food quality tests are required. The presented experimental research confirms the overall positive influence of NTP treatment, even for very low treatment times, on wheat flour and its products. The presented findings are significant for the potential implementation of this technique on an industrial level.

## 1. Introduction

Plant-based food, such as cereals, grains, and their products, are an important source of energy for humans and animals due to their constituents, i.e., carbohydrates, proteins, vitamins, fats, and minerals [1]. In the human diet, grains are usually present in powder form, i.e., as flour, produced by grain milling and its products.

Food processing is a crucial step in the food supply chain of the modern food industry, which ensures food safety and intensifies food functional properties (swelling capacity, water absorption, elasticity, gas retention, gelatinization temperature, bulk density, shelf life, etc.) [2,3,4]. The processing of plant-based food is mainly categorized into primary (starting immediately after harvesting, which ensures ease of transportation) and secondary processing (conversion into different food products, improving functionality, shelf life, etc.) [5]. Thermal processing (including drying and evaporation) of food is a common and simple yet effective method that uses heat to restrict microbial activity, and in this way reduces spoilage and increases a product’s shelf life. However, it can seriously reduce food quality, e.g., color, texture, flavor, freshness, and nutrient value [6,7]. Other conventional food sterilization and processing techniques, e.g., chemicals [8,9], high pressure [10], microwaves [11], ultra-sonication [12,13], bio-preservatives [14], and vacuum processing [15], are also being practiced, but their influence on food quality preservation and retention have its drawbacks.

Modern lifestyles and the aspiration towards healthy eating have created a demand for minimally processed food, fewer synthetic additives, clean-label products, and highly natural ingredients [16]. Sustaining the equilibrium between the necessities for intensive food processing prior to intake and consumer demand for minimally processed food is challenging due to the risk of foodborne illness. This situation indicates the need for novel food processing methods, which would help in obtaining a balance between the minimal food processing necessary to enhance food functional properties and food safety.

Wheat is ranked as the prime staple crop that ensures food and nutritional security [17]. Chemically, the whole wheat grain is composed of starch (≈58%), protein (≈11%), non-starch polysaccharides (≈13%), lipids (≈2%), minerals (≈2%), and water [18]. Wheat proteins are traditionally classified according to their solubility properties, i.e., albumin (soluble in water), globulin (in a salt solution), gliadin (in 70% ethanol), and glutenin (in acid or alkali) [19]. Gliadin and glutenin (about 85% of the total proteins), also known as gluten proteins, contribute to special functional properties of dough (viscosity, extensibility, gas retention, cohesiveness properties, etc.) [20,21,22,23] and allow wheat to be processed into a wide range of products, including bread, cakes, pasta, noodles, etc. [24,25,26]. All the wheat varieties developed for broad-acre agriculture share similar gluten proteins in their structure [25,27,28].

In the wheat dough formation process, gluten proteins are hydrated and are transformed into a continuous cohesive visco-elastic gluten protein network, accompanied by an increase in the extractability of gluten proteins [29]. The functionality of these proteins in a dough strongly depends on several factors such as dough recipe, water temperature, enzymes, etc. [30]. Dough network formation is affected by the formation, number, and distribution of intermolecular Sulphur–Sulphur or disulfide (S-S) bonds between gluten protein networks [31]. The amino acid cysteine and its oxidized form cystine are naturally found in wheat flour and play an important role in the formation and termination of S-S bonds [32]. The formation of covalent bonds via disulfide linkage starts during the kneading process, where cysteine residues (an S-H containing amino acid) react with each other (oxidation of reactive S-H groups to cystine) to form S-S bonds [33,34,35]. The weakening of dough starts with the presence of a free cysteine, which reduces single S-S bonds to two –SH bonds [32].

The processing of wheat flour is a common practice on an industrial level, not only to improve its hygiene, safety, textural properties, and shelf life of products but also to affect the dough strength and weakening agents according to product specifications. In the food industry, chemicals such as ascorbic acid, azodicarbonamide (ADA), and potassium bromate are used as dough properties enhancers [36]. For example, ADA, patented in 1959, is one of the fastest oxidants used as a dough conditioner that reduces the content of free –SH groups in the dough, hence promoting the S-S bond networking [37,38]. Ozone, which is a topic of extensive research [39,40,41], can be used as an alternative to chemicals for improving wheat dough properties, as it is termed “generally recognized as safe” (GRAS) [42,43]. Its strong oxidizing potential, clean nature, i.e., only oxygen remains after the treatment, and in situ generation by an electrical discharge, make ozone an ideal substitute for many chemicals in food processing [44].

One promising approach to food treatment is the treatment with non-thermal plasma (NTP). Plasma, representing the fourth state of matter, is an overall neutral, ionized gas consisting of ozone in abundant amounts, along with other active species (positive and negative high-energy particles, electrons, and neutrons) [45,46,47,48]. Contrary to ozone generators, NTP ozone is produced in situ, together with other active species. An electron temperature and a thermodynamic equilibrium inside an ionized gas are the decisive factors for plasma classifications [49]. In thermal plasma, electrons and bulk gas molecules are in the state of thermal equilibrium, which contributes to very high plasma gas temperatures [50,51], thus not suitable for thermally sensitive applications, i.e., food. Such a thermodynamic equilibrium is lacking in NTP, as the electron temperature of the gas is much higher (~10^5^ K) than of the gas bulk molecules (~300 K) [52,53]. Atmospheric plasma jet, low-pressure glow, microwave discharge, laser-produced plasma, and dielectric barrier discharge (DBD) are a few examples of NTP types. However, DBD is widely used due to its simple formation, non-thermal nature, and production of abundant amounts of ozone.

Lately, NTP has gained much attention in food applications due to promising results in impacting quality attributes, food preservation, safety, and microbial inactivation [54,55]. The interaction between different types of NTP with wheat grains and flour and the effects on their functional properties have been investigated [56,57,58,59,60]. Bahrami et al. [56] and Mishra et al. [61] reported an increase in visco-elastic properties of wheat dough after the NTP treatment of flour. NTP treatment was reported to lead to starch modifications that significantly alter the dough viscosity [62]. Some studies showed that the NTP treatment did not affect the total protein count; however, a trend towards conversion to higher molecular weight proteins was determined, together with changes in surface morphology [61,63]. However, the literature does not offer detailed information on the effects of specific NTP types on wheat flour constituents, dough, and baked products.

The goal of the present study is to investigate in detail the influence of the DBD type of NTP on wheat flour functionality and the properties of its products. NTP-type DBD was chosen due to its low, controllable temperature level and abundant amount of ozone production, along with other active species. To evaluate the potential of this method for an industrial application, very short treatment times (1, 3, and 5 min) were applied. The analysis was based on evaluating the changes in properties of flour (moisture content, protein, starch, color, microbial activity, enzymes, and fat amount), dough (visco-elastic properties, starch, wet and dry gluten, and water absorption), and baking product properties (color, freshness, baked volume, crumb structure, softness, and elasticity). Based on the detected positive and negative aspects of the NTP treatment, the applicability of this method for wheat flour treatment was discussed.

## 2. Materials and Methods

In this research, Germany’s wheat flour type 550 (Rettenmeier GmbH Kunstmühle, Horb am Neckar, Baden-Württemberg, Germany), equivalent to all-purpose flour, was used for treatment, preparation of dough, and baking products (test bread). Flour, dough, and baked products were analyzed before and after the NTP treatment.

### 2.1. NTP Treatment Experimental Setup

**Figure 1:**
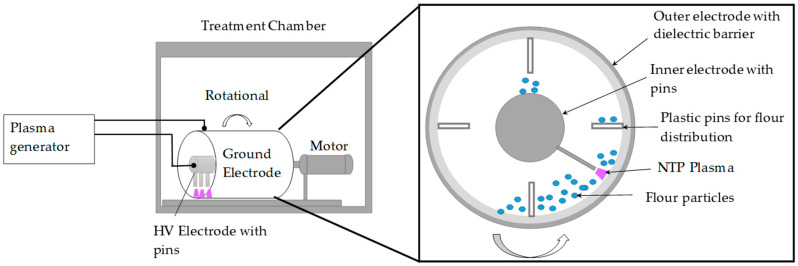
Experimental setup for Non-thermal plasma (NTP) treatment of wheat flour.

The experimental setup (Figure 1), which was used for the flour treatment, consisted of three main parts: (1) a high-voltage power supply (HVG80–3000, company Diener, Ebhausen, Germany) for NTP generation, (2) a cylindrical, rotational reactor, where flour was treated by in situ produced NTP, and (3) a data acquisition (DAQ) system.

A high-voltage power supply (*P* = 120 W, *f* = 80 kHz, *U* = 7 kV) was used to generate the DBD plasma (shown as purple color in Figure 1) inside the reactor. Flour was placed inside a cylindrical, rotational batch reactor (shown as blue color dots in Figure 1), which was open to the atmosphere, in order to avoid the formation of combustible/explosive dust. The reactor consisted of two concentric electrodes:

(1) the outer (ground) electrode, in the form of a hollow cylinder (*D* = 150 mm, *L* = 300 mm) made of stainless steel and mounted axially on a rotating shaft. To stop the formation of arcs, a dielectric barrier (thickness = 2 mm) made of Plexiglass was attached to the inner side of this ground electrode.

(2) the stationary, inner (high-voltage) electrode is positioned coaxially to the ground electrode. A row of aluminum pins (*H* = 43 mm, thickness = 5 mm) was attached to the high-voltage electrode to reduce the discharge gap to 2 mm and to reduce the overall power needed to generate DBD plasma inside the reactor. The uniform flour treatment inside the reactor is ensured by the presence of plastic baffles (*H* = 30 mm, thickness = 3 mm). Further details of the setup can be found in our previous publication [64].

### 2.2. Dough Preparation

Doughs were prepared from untreated and treated flour samples in order to identify and compare the changes induced by the NTP. Flour (3000 g), water (60% i.e., 1800 g), salt (60 g), dry yeast (75 g), fat (30 g), and sugar (30 g) were mixed in a dough-preparing machine (SP 12–DIOSNA spiral kneading machine, DIOSNA Dierks & Söhne GmbH, Osnabrück, Germany). The kneading process included a mixing phase (at 25 Hz mixing frequency) of 2 min, followed by a kneading phase (at 50 Hz) for 6 min. After kneading, the dough was left to rest for 10 min, followed by a proofing step. The dough was divided into 6 parts weighing 600 g each. Three of them were left for normal proofing and the rest for extended proofing. Normal proofing was performed for 60 min (90 min in case of extended proofing) at 32 °C and 85% relative humidity. Afterward, the dough was used for a series of analyses.

### 2.3. Baking Parameters

Six sandwich breads were prepared to observe the baking properties. They were baked free and settled on a baking plate (top-bottom heating at 210 and 220 °C, respectively) in an electric oven for 30 min. They were left to cool for 10 h at room temperature prior to baking analysis.

### 2.4. Analysis of Flour, Dough, and Baked Products

#### 2.4.1. Flour Hydration Analysis

Moisture content was determined three times per sample, according to the standard method (ICC Nr. 110/1) approved by the International Association for Cereal Science and Technology. Based on these measurements, the standard deviation was calculated.

The variation in the water activity (a_w_-values) of the flour samples treated by NTP was obtained using the sorption isotherm method [65]. The analysis was repeated three times, based on which the standard deviation was obtained.

Near-infrared (NIR) spectroscopy is a rapid, accurate, and non-destructive technique that is commonly used in food analysis using the interaction between electromagnetic energy (in the range of 800–2500 nm) with the matter in question [66]. It was used to determine wheat flour quality parameters, e.g., moisture, protein content, ash, water absorption, etc. [67]. Contrary to the conventional water content analysis, where a sample is heated to 130 °C, NIR does not need heating. The standard deviation of this measurement set was determined based on three measurements per sample.

#### 2.4.2. Flour Protein Analysis

In the present research, a standard method for determining gluten (ICC Nr. 155) was used to quantify the wet and dry gluten content and the gluten index (GI). The GI is correlated with the protein strength, which is calculated based on the percentage of wet gluten that remained on a sieve (84 microns for wheat flour) after centrifuging [68]. The analysis was repeated four times, based on which the standard deviation was calculated.

Sedimentation analysis is a physicochemical test that provides information about the protein swelling capacity, which directly affects the baking quality (e.g., loaf volume) of a wheat flour dough. This analysis involves the absorption of water and the swelling of protein in a water-flour, lactic acid, and isopropyl alcohol slurry. The absorption capacity is dependent on the protein quality [69]; higher protein quality leads to higher water absorption, which reduces the protein particle compactness, hence reducing the density and leading to lower sediment velocities of protein particles and vice versa. The sedimentation analysis (according to Zeleny) was performed using a standard method (ICC Nr. 116/1) and repeated two times to calculate the standard deviation.

The adhesive properties of wheat flour were analyzed by kneading gluten balls ten times in a 2% indium, sodium chloride, and water solution for one hour.

The farinograph analysis was performed according to the standard method (ICC Nr. 115/1). Dough quality is additionally characterized by dough stability. This is the ability of the shaped piece of leavened dough to maintain its shape during the proofing phase, which can be estimated by a farinograph. Stability is connected to the tolerance of flour to kneading and overmixing, and it is measured as the time during which a dough sustains its maximum consistency. It corresponds to the difference between the dough development time (i.e., the point at which the top of the farinograph curve intercepts the 500 BU line) and the departure time (i.e., the point where the top farinograph curve leaves the 500 BU line) on a farinograph chart. Dough softening is the difference between the heights of the farinograph curve between the point of the maximum resistance to mixing and the point measured 12 min afterward. Softening is concerned with the extensibility of dough. Flour that is too hard (higher protein content or S-H bonding) needs softening agents to break down the dough’s strength in order to meet the handling and similarity in baked products on an industrial level. The analysis was repeated two times, based on which the standard deviation was calculated.

Dough’s resistance to stretch and gluten protein extensibility was analyzed by an apparatus, “Kieffer rig” [70]. This apparatus can be connected to any material testing machine to measure the stretch force by adjusting the stretch speed and, finally, the temperature to calculate the offered resistance to stretchiness or extension and capability of extensibility (distance to break) of wheat dough [71]. The kneading was done in Perten Glutomatic for 3 min, and the dough rest time was 10 min. The test was repeated 11 times, based on which the standard deviation was calculated.

#### 2.4.3. Other Flour Quality Analysis

The Falling number (FN) records the activity of α-amylase, which is responsible for the gelatinization and breakage of starch granules. α-amylase is an enzyme that breaks down a long-chain starch to simple sugars, which in the end, impacts the quality of baked products. The Perten-Hagberg number is the measure of the time (in seconds) required to travel a certain distance for a stirrer through a hot water-flour slurry [72]. If the stirrer falls slowly (higher FN number), the structural integrity of starch chains is preserved, i.e., it is not destroyed by α-amylase activity, and vice versa. FN was performed using the ICC Nr. 107/1 and repeated six times to calculate the standard deviation.

The gelatinization and pasting behavior of wheat flour can be studied by amylograph, which provides an easy and standardized performance and an effective correlation with the final product quality [73]. The gelatinization, or the peak temperature, indicates the temperature at which swollen starch granules in water dispersion under a shear force tend to disintegrate from a crystalline state to a gel [73]. Accordingly, the starch viscosity at this temperature is called gelatinization or peak viscosity. At this viscosity, a balance between granules’ swelling and granules’ breakage is reached shortly before their physical breakdown. The test was done according to the ICC Nr. 126/1. The tests involved the heating of samples from 30 to 95 °C at a constant heating rate of 1.5 °C/min. The starch gelatinization temperature, the viscosity change, and the starch damage were then analyzed based on the experimental findings. The tests were repeated two times to calculate the standard deviation.

#### 2.4.4. Color Analysis of Flour and Products

In 1976, the International Commission on Illumination (CIE) recommended a method CIE L*a*b color space that expresses color numerically and can be used for precise color communication [74]. The color of flour and products was analyzed by the color measurement device (Konica Minolta, Konica Minolta Business Solutions Deutschland GmbH, Langenhagen, Germany). In this method, the *L* coordinate represents the lightness (0 = black, 100 = white), the *a* coordinate indicates the red to the green range, and the *b* coordinate indicates the yellowness. E* represents color uniformity, and Δ*E* shows the difference in colors. The analysis was repeated three times, based on which standard deviation was calculated.

#### 2.4.5. Baked Product Analysis

Changes in starch and protein functional properties due to plasma treatment also affect the baking product properties. Starch and gluten interact to form a stable network that entraps the fermentation gas and resists a bread collapse during the baking and cooling stage [75]. Starch damage, which is regulated by amylase activity, increases the water absorption capacity of flour [29]. This moisture gradient affects the starch gelatinization and swelling in a dough, thus affecting the dough’s softness and resilience properties [29,76].

To analyze the effects of NTP treatment on the baking product, the dough was prepared as presented in Section 2.2. Six equal size (600 g) sandwich breads were made, three from normal proofing time, i.e., 60 min, and three from extended proofing time, i.e., 90 min. The apparent bread volume was determined by the Rapeseed displacement method. The texture analyzer, based on the American Association of Cereal Chemists (AACC) bread firmness method, was used to evaluate the bread’s freshness. Within this test, the resilience, i.e., the ability of a pastry product to absorb energy when deformed elastically and to release it upon unloading, was determined.

#### 2.4.6. Microbiological Analysis

Wheat grains and flour can carry different contaminants from the environment as they are raw agricultural products. Microbial contaminants are mostly present on a grain surface, which are mostly removed during the milling process. However, flour can still contain dangerous contaminants [77]. Enterobacteriaceae such as Serratia, Pantoea, Escherichia, Enterobacter, and Raoutella are commonly listed microorganisms in flour microbiota. It is usually considered by food manufacturers as a hygiene indicator to monitor the effectiveness of the implemented preventive measures [78].

The presence of the microorganisms sulfite-reducing clostridium, yeast, mold, E.coli, Salmonella, Lactobacillus, Staphylococcus, Bacillus cereus, and Enterobacteriaceae in flour was analyzed according to the German standard DIN ISO 21528-1/2.

### 2.5. Experimental Procedure

Before the treatment, the NTP reactor was thoroughly cleaned, and 150 g of type 550 flour was added to it. The reactor rotated at a constant speed of 10 RPM to ensure uniform flour-plasma contact. A high-voltage generator was operated at the maximum power (120 W). A maximum of 3000 g of plasma-treated flour was needed for flour, dough, and baked products analysis. Therefore, twenty batches of 150 g were treated in the batch reactor and mixed together in a vessel (SP 12–DIOSNA spiral kneading machine) before their use in the respective analysis.

## 3. Results and Discussion

In order to investigate the effects of NTP on wheat constituents, the analysis was performed on untreated and treated wheat flour and corresponding wheat dough and wheat baked products. Following section lists and encloses the performed analysis in detail.

### 3.1. Influence on the Flour Hydration Properties

Quantification of the amount of water is the first step in reporting the wheat quality attributes, especially the hygienic conditions. A variation in the amount of water within the wheat flour directly influences the flour’s stability and supports chemical reactions. Flour moisture content also affects its transportation properties, such as flow from silos, long-term transportation, compression, mixing, and packaging. Water content significantly affects the formation of liquid bridges and the capillary forces acting between the flour particles [79]. This part of the research encloses the analysis of free and bound water content in untreated and plasma-treated wheat flour samples by different analytical techniques.

#### 3.1.1. Moisture Content Analysis

**Figure 2:**
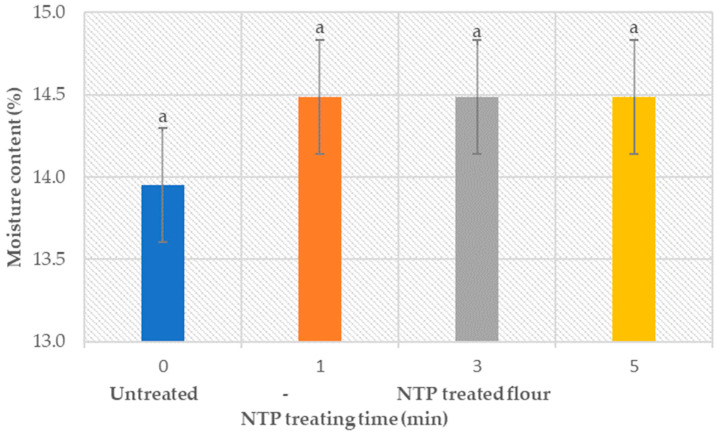
Moisture content of the untreated and treated wheat flour (type 550) samples at different plasma treatment times with analysis of variance (letters with alike are not significantly different).

Moisture content accounts for the bound and the free water present in flour. The dependency of the moisture content of wheat flour on the treatment time (1, 3, and 5 min) is shown in Figure 2.

Obtained results show a change of less than 1% in the moisture content of the treated samples with respect to the control samples. According to the literature [80], the moisture content between 12 and 15% keeps the flour stable at room temperature. A slight increase in the moisture content of the treated samples indicates the surface modifications of flour particles due to NTP. The literature reports that the plasma treatment results in a decrease in water contact angle, hence supporting the high permeability of water [81]. Obtained results confirm that the moisture content of plasma-treated flour samples stays in the range where flour stays stable at room conditions, i.e., 12–15%. However, these results must be taken with a reserve, as the *p*-value obtained by analysis of variance (ANOVA) is greater than 0.05 (provided in the Supplementary Materials), indicating the lack of significant statistical difference existence between the samples.

#### 3.1.2. Water Activity Analysis (a_w_–Value)

The moisture content itself is not enough to predict the safety, stability, and shelf life of a product. The amount of free water available to support chemical or biological processes in flour, which determines the potential growth capability of microorganisms, is expressed by the water activity analysis in terms of the a_w_-value. The optimal value range of the water activity (0.4–0.7) limits flour spoilage by microbial activity, also reported by Labuza et al. [82].

**Figure 3:**
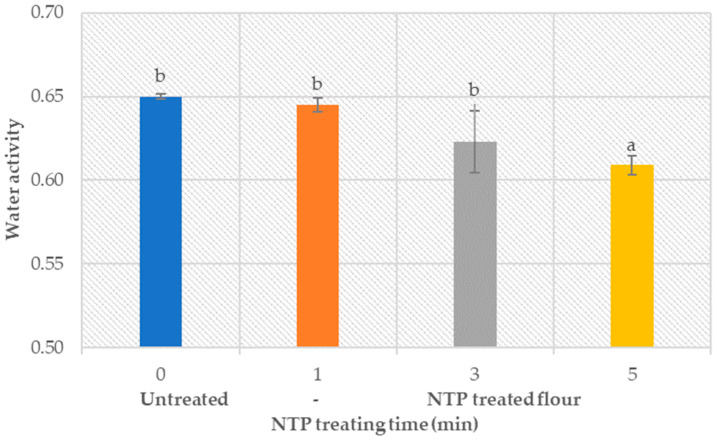
Water activity value (a_w_-value) of the untreated and treated wheat flour (type 550) samples at different plasma treatment times, measured by the sorption isotherm method.

Obtained results (Figure 3) demonstrate a decline in the water activity of the treated wheat flour with the increased NTP treatment time. The a_w_-value remained in the optimal a_w_—range for the tested treatment time, thus ensuring the flour’s safety and stability during storage at room temperature. The ANOVA analysis (*p* < 0.05) (provided in the Supplementary Materials) confirms the existence of significant statistical differences between untreated and treated samples, i.e., the effect of NTP treatment on flour properties was statistically significant.

#### 3.1.3. Near-Infrared Spectroscopy for Moisture Content Analysis

The findings of NIR spectroscopy also confirmed that NTP treatment slightly decreased the water content in wheat flour; however, it stayed close to 14%. Obtained results also coincide with the findings presented in Figure 3 (therefore not shown separately), where the water activity is shown to decrease with the plasma treatment times.

All three analysis techniques lead to the conclusion that NTP does not significantly alter the free and bound water content of wheat flour, which stays at the optimal, permissible level to sustain its stability and promote chemical reactions at a later stage.

### 3.2. Flour Protein Properties

Wheat proteins (11.1 g/100 g flour type 550), especially gluten protein (85% of the total protein amount), are important constituents of wheat flour that play a significant role in dough network development through the formation of disulfide bonds within protein molecules. They also have an important impact on the baking product quality due to their effect on the water absorption capacity, cohesivity, viscosity, and elasticity of a wheat dough [68]. The following part of the research encloses the effects of the treatment time on the wheat proteins through different protein analysis techniques.

#### 3.2.1. Gluten Content (Wet and Dry) and Gluten Index

The quantity and quality of gluten proteins are considered one of the major quality parameters for wheat flour. It has a major impact on the cooking quality, e.g., appearance, loss of solids in cooking water, and texture (crumb structure, thickness, and adhesiveness) [83]. The effects of gluten on baking properties can be found elsewhere [84]. The wet and dry gluten content expresses the amount of gluten present in flour, which depends on the genotypes, intensity of breeding as fertilizer dose, and pedoclimatic conditions [85]. Table 1 lists the determined gluten quantity and quality parameters of untreated and treated flour samples.

Obtained results revealed that the wheat gluten content (dry and wet) of samples treated up to 3 min is comparable to the control sample, i.e., no significant change in the total protein level of wheat flour was noticed (ANOVA analysis, provided in the Supplementary Materials). This was also confirmed by Bahrami et al. [56]. However, further treatment (up to 5 min) results in lowering the gluten (dry and wet) amount. Negative effects of longer NTP treatment (45 min), which, i.e., deteriorates the hydration capacity of gluten, were reported elsewhere [86].

The GI, as a quality attribute of wheat gluten, showed a progressive increase with the NTP treatment time. It reached the maximum value of 97.2 ± 1.6, corresponding to an almost 7% increase in comparison to untreated wheat flour. A number of studies [56,61,86] confirmed the contribution of NTP in enhancing the disulfide bonds between the glutenin subunits, thus leading to significant changes in the distribution of higher molecular weight glutenins and to a higher GI.

#### 3.2.2. Zeleny’s Sedimentation Analysis and Adhesive Properties Analysis

Results revealed that plasma treatment did not change (*p* > 0.05) (provided in the Supplementary Materials) the sedimentation values. However, the obtained sedimentation values of 36 ± 1 mL confirm the presence of medium flour (type 550) under investigation.

#### 3.2.3. Water Absorption: Farinograph Tests

Farinograph analysis is used to observe dough behavior during the kneading process. Dough attributes are dependent on the water absorption capacity and can be determined by the amount of water that is used to reach the standard consistency (500 BU line of Farinograph) [87,88]. Water absorption plays an important role in dough network building, as it provides a ground for chemical reactions to occur during the dough development step.

Table 2 shows the water absorption capacity of doughs made from untreated and treated flour obtained by the Farinograph test.

Treated dough samples made from treated and untreated flour show the same water absorption behavior, which can be also confirmed by the *p*-value (>0.05) obtained by ANOVA analysis (provided in the Supplementary Materials). Depicted results lie in the standard range (>58–60%) for the water absorption capacity of the wheat flour, confirming no negative effects of NTP on wheat flour water absorption capacity.

#### 3.2.4. Dough Stability and Softening: Farinograph

Results shown in Table 3 demonstrate the ability of NTP to intensify the dough stability, which accelerates with the treatment time (*p* < 0.05, ANOVA, provided in the Supplementary Materials) and reaches the maximum value (i.e., above 15%) at the treatment time of 5 min. Results concerning the softening properties indicate a trend towards a decline in softening properties (i.e., higher dough strength) with NTP treatment. The change in these properties is connected to the change in gluten proteins, as described in Section 3.2.1.

#### 3.2.5. Dough Stretch Resistance, Dough Extensibility, and Their Ratio: Kieffer Rig

Table 4 encloses the results for the stretch resistance, extensibility, and their ratio for dough samples, prepared by untreated and treated flour as a function of treatment time. The *p*-value (<0.05) obtained by ANOVA analysis (provided in the Supplementary Materials) confirms the existence of a significant statistical difference between the control and the NTP-treated samples.

The results demonstrate that the dough strength or firmness increased for samples prepared from treated flour, in comparison to the control samples, i.e., dough produced from untreated wheat flour. The maximum change in dough extensibility properties and resistance to elongation were observed at the treatment time of 3 min, which corresponds to an increase of 30% and 36%, respectively.

The variation (increase or decrease) in wheat flour-dough protein properties is strongly related to the end product specifications. Thus, it is not easy to claim the extent of positive or negative change in protein properties due to NTP treatment. For example, due to the formation of –SH bonds, the dough is stronger, meaning that the handling of the dough is good and it can better retain its structure. On the other hand, loaf strength can negatively affect the volume of the baked bread while CO_2_ release faces more resistance, and thus, the crumb volume might be lower.

### 3.3. Other Flour Quality Analysis

Starch, which is a pure carbohydrate, represents the major part (63–72%) of the wheat grain consisting of glucose polymers, amylose, and amylopectin [89]. Two types of starch granules, type A (long and lenticular shape) and type B (small and spherical), are identified in wheat flour [90]. Scanning electron microscopy (SEM) images of starch types can be found in our previous study [64].

Starch quality and quantity directly affect the wheat dough’s strength, as starch is physically entrapped in a gluten network and acts as an inert filler during dough making [89]. In baked goods, starch is responsible for pasting properties due to its viscosity change during heating and water absorption [91]. In this section, the results of starch component analysis before and after the NTP treatment are presented.

#### 3.3.1. Falling Number

The FN numbers of untreated and treated flour samples were compared. Obtained results showed a reduced α–enzymatic activity, i.e., a higher FN number (>300) for all the samples. The *p*-value obtained by ANOVA analysis (provided in the Supplementary Materials) was above 0.05, representing no significant statistical difference between the control and plasma-treated samples. Hence, it was concluded that NTP does not affect the structural integrity of the starch chain.

#### 3.3.2. Starch Gelatinization Temperature, Viscosity, and Starch Damage

Obtained results show no significant change (*p* > 0.05, provided in the Supplementary Materials) in the pasting properties of starch after the plasma treatment.

### 3.4. Color Properties of Flour and Baked Products

The color of flour and baked products is an important quality attribute that can be evaluated by human vision and by instruments. In the current research, the CIE criterion was used to evaluate the influence of plasma treatment on the color of flour and baked products.

#### 3.4.1. Color Analysis of Flour

The values of color grade for control wheat flour confirmed its color close to white. Obtained results show that the color grades of the treated samples remain close to the untreated sample (>90 for L and >10 for b). This confirmed no negative effect of the NTP treatment, i.e., the color of flour particles remained unchanged and uniform.

#### 3.4.2. Color Analysis of Baked Products

The CIE color coordinates values were also obtained for baked products made of untreated and NTP-treated flour and are summarized in Table 5. In contrast to the findings regarding the wheat flour samples, a change in the color of the wheat-baked products produced from treated flour was observed. The values of *L*, *b*, *a*, and Δ*E* showed that plasma caused a lighter color in the baked wheat products.

### 3.5. Baking Product Quality

In the presented research, the NTP effects on the baked product, i.e., bread volume, its softness, extension, and aroma properties, were investigated.

#### 3.5.1. Product Volume

**Figure 4:**
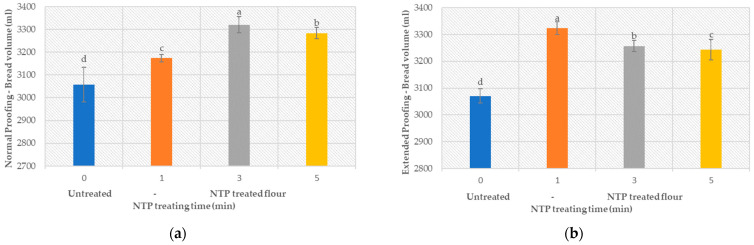
Effect of NTP treatment of flour on the bread volume (**a**) normal proofing time (**b**) extended proofing time.

Figure 4 shows the volume of sandwich bread produced from untreated and plasma-treated flour at two different proofing times, i.e., (a) 60 min and (b) 90 min. In both cases, plasma treatment clearly resulted (*p* < 0.05) (ANOVA analysis, provided in the Supplementary Materials) in a higher loaf volume, i.e., on average, 9%. This effect is connected to the influence of plasma on the protein and the starch network formation, which were addressed in Section 3.2 and Section 3.3.

Studies [92,93] indicate that an increased proofing time can result in an increased loaf volume. Figure 4b shows that the extended proofing time (90 min) also significantly changes (*p* < 0.05) the dough volume when the flour was treated with plasma. However, at the treatment time of 1 min, a maximum increase of 5% in volume was observed. This corresponds to the finding shown in Section 3.2 and Section 3.3, where an increased protein-starch network formation was noticed at higher treatment times. The dough is more stable and resists restoring its form on the production of CO_2_ gas by yeasts.

#### 3.5.2. Product Freshness

**Figure 5:**
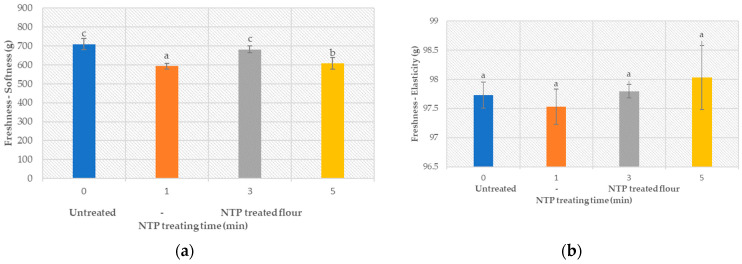
Effect of the NTP treatment on the bread freshness, expressed in terms of (**a**) softness and (**b**) elasticity.

The texture and pliability of bread are indicators of freshness and can be evaluated through texture analysis. Figure 5 shows the measured freshness of products, expressed through the softness and the elasticity of the tested bread. Results indicate softening of bread produced from NTP-treated flour (with *p* < 0.05, provided in the Supplementary Materials), which could be related to increased visco-elastic properties of the dough (increase in GI, Table 1). On the other hand, the elasticity of bread samples was not significantly affected (provided in the Supplementary Materials) by increased plasma treatment times (*p* > 0.05).

#### 3.5.3. Sensory Analysis

Sensory analysis is vital for food products, as it establishes the quality and the acceptance of a product among defined target customer groups and markets. The sensory organs (eyes, nose, mouth, skin, and ears) are applied to analyze and evaluate the food product characteristics according to various schemes. DLG (Deutsche Landwirtschafts-Gesellschaft, i.e., German Agricultural Society) is one of the oldest institutions in Germany that provides certified sensory tests for food and beverages [94]. Ozone, which is extensively produced during the NTP treatment, is known for its specific smell. Therefore, treated flour and corresponding baking product were tested for smell and taste using the DLG—sensory analysis scheme. The obtained results excluded the retention of ozone smell in the flour and baked products, which is beneficial for the future application of NTP in wheat flour treatment.

### 3.6. Flour Microbiology (Bacteria and Enzymes)

**Figure 6:**
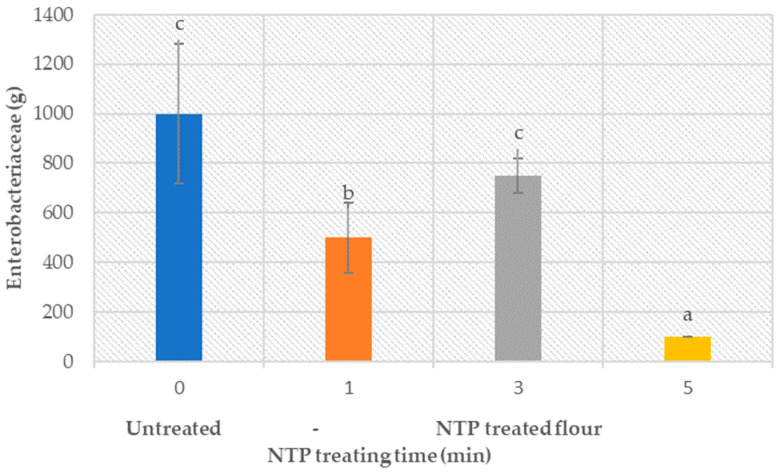
Effect of plasma treatment times on the Enterobacteriaceae microorganism count.

The studied flour was free from most of the detrimental microbial colonies; therefore, no reduction of the overall microorganism activity due to plasma treatment could be established. However, plasma treatment did result in a statistically significant reduction of Enterobacteriaceae (*p* < 0.05, provided in the Supplementary Materials), as shown in Figure 6. The presented results demonstrate the ability of NTP treatment to reduce the microorganism count between 20 and 80%.

### 3.7. Flour Fat Amount

The function of fat is versatile during bread making. The chemistry and the physical properties of fat impart richness and tenderness to improve flavor and mouth-feel properties [95]. The role of fat in enhancing the aeration of dough by CO_2_ and supporting the role in leavening and volume of baked products was also reported in the literature [96,97].

**Figure 7:**
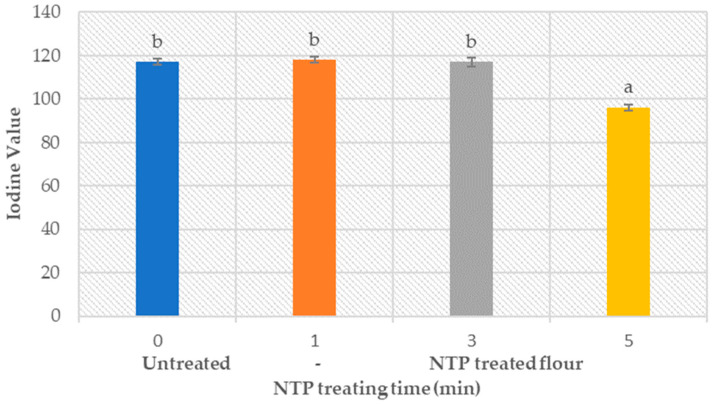
Effect of NTP treatment on the fat amount in untreated and treated wheat flour, expressed through the iodine value.

An amount of unsaturated fat, present in both untreated and treated flour samples (Figure 7), is represented as the iodine value (IV), which shows the mass of iodine consumed by 100 g of fat.

Analysis showed that the NTP treatment of up to 3 min did not change the fat amount in the flour. However, the treatment time of 5 min resulted in an 18% reduction of fat amount in wheat flour. This means that short plasma treatment times do not deteriorate the properties induced by fat in the baked products; however, it reduces the amount of fat in the treated flour at higher plasma treatment times.

### 3.8. NTP Treatment vs. Ascorbic Acid

In order to compare the impact of flour improver (ascorbic acid (ASC), glucose oxidase (GOX)), and the NTP treatment on flour properties, the extensographic properties were emphasized rather than the farinographic. In this study, ASC and ASC + GOX were added (0.2%) to untreated flour samples to compare their enhancement properties with the potential of NTP treatment.

**Figure 8:**
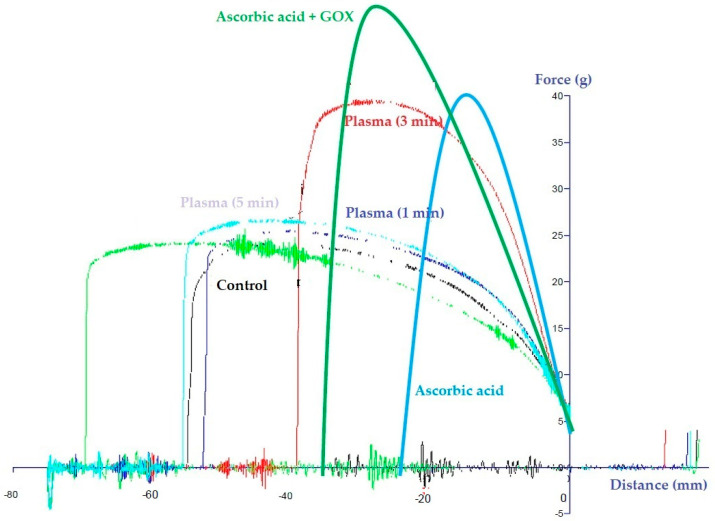
The extenographic properties of control, plasma treated, and ascorbic acid flour samples.

Obtained results (Figure 8) depict that the 3 min plasma treatment resulted in a similar extenographic properties enhancement of flour as induced by the ASC. On the other hand, it did not reach the extenographic properties enhancement of ASC + GOX. Furthermore, too short or too long NTP-treatment did not enhance extenographic properties in comparison to the control sample.

This finding indicates that the proper NTP treatment of flour could significantly affect the flour’s extenographic properties and, in this sense, would be able to replace chemicals for this purpose. However, further investigation in terms of the optimal treatment time is needed.

## 4. Conclusions

The overall conclusion, which can be drawn from the presented experimental analysis, is that the NTP treatment of wheat flour showed positive effects on flour properties and properties of its products, even for very short treatment times (≤5 min). Plasma species produced during NTP treatment showed a potential to affect the flour and dough structural network attributes, i.e., protein, starch, color, surface structure, enzymatic, and microorganisms activity to a certain extent. An important finding is that no negative effects on the product quality were observed.

NTP treatment made treated flour slightly moister, which is expressed as an increase in the moisture content, but the amount of free water, expressed as the water activity, decreased. Decreased water activity (<0.7) is expected to result in improved flour stability and product shelf life. For the treatment duration of 5 min, flour protein strength (expressed through the GI) increased by 8%, which is an indicator of a more stable dough. The NTP treatment of 3 min resulted in an increased dough extensibility and resistance to elongation, i.e., an increase of >30% and >45%, respectively.

Regarding a baking product, an increase in dough volume of, on average, 9% was observed for the loaves made from plasma-treated flour. This finding indicates the softening of a breadcrumb without causing a change in the bread elasticity. Even though the color of treated flour samples stayed unchanged, the whiteness of a breadcrumb was increased (>6%). The enzyme activity within flour decreased, which has a positive effect on safety; however, α amylase activity is reduced, which is essential for the dough’s softness. Longer treatment times (5 min) resulted in a decreased amount of fat in flour, which may reduce the flavor and mouth-feel properties of the baked goods.

Presented results indicate that the NTP treatment of flour could be used as an alternative to chemicals for changing the flour and respective dough functional properties on an industrial scale. However, as an outlook to this work, the upscaling of this technology can be challenging in terms of the reactor design to provide a uniform, direct contact between flour and plasma species, as the optimal flour treatment procedure is vital for further implementation of NTP-based flour treatment. Even though some aspects of food safety were investigated, the applicability of NTP treatment in the food industry still needs to be approved, e.g., a review of the processes with regard to the Novel Food Regulation is suggested.

## Figures and Tables

**Table 1:** Effects of NTP treatment times on the gluten content and the GI of wheat flour samples.

Samples		Wet Gluten (%)	Dry Gluten (%)	GI
Untreated Flour	Control	23.9 ± 0.5 ^b^	10.3 ± 0.2 ^b^	89.4 ± 3.3 ^b^
Treated Flour	1 min	23.8 ± 0.5 ^b^	10.3 ± 0.1 ^b^	89.6 ± 5.5 ^b^
	3 min	24.3 ± 1.5 ^b^	10.5 ± 0.0 ^b^	90.9 ± 3.3 ^b^
	5 min	22.1± 0.4 ^a^	09.9 ± 0.1 ^a^	97.2 ± 1.6 ^a^

**Table 2:** Water absorption capacity of dough samples made from untreated and treated wheat flour and measured by Farinograph.

Samples		Farinograph (%)
Untreated Flour	Control	60.5 ± 0.1 ^a^
Treated Flour	1 min	60.5 ± 0.2 ^a^
	3 min	60.4 ± 0.2 ^a^
	5 min	60.4 ± 0.1 ^a^

**Table 3:** Stability and softening of dough samples prepared from untreated and treated wheat flour andmeasured by Farinograph.

Samples		Stability (min)	Softening (BU)
Untreated Flour	Control	7.13 ± 0.1 ^d^	53 ± 0.1 ^d^
Treated Flour	1 min	6.96 ± 0.2 ^c^	55 ± 0.2 ^c^
	3 min	7.60 ± 0.2 ^b^	52 ± 0.1 ^b^
	5 min	8.26 ± 0.15 ^a^	49 ± 0.4 ^a^

**Table 4:** Stretch resistance and extensibility of doughs made of untreated and treated flour samples and measured by the Kieffer rig.

Samples		Stretch Resistance (R_max_) (g)	Extensibility (E) (mm)	Ratio (R_max_/E)
Untreated Flour	Control	24.6 ± 2.6 ^b^	48.4 ± 5.2 ^b^	0.5 ± 0.1 ^b^
Treated Flour	1 min	26.3 ± 1.5 ^b^	45.3 ± 4.7 ^b^	0.6 ± 0.1 ^b^
	3 min	36.0 ± 3.8 ^a^	32.2 ± 3.2 ^a^	1.1 ± 0.2 ^a^
	5 min	24.3 ± 1.6 ^b^	45.8 ± 4.7 ^b^	0.5 ± 0.1 ^b^

**Table 5:** Color analysis of wheat-baked products produced from untreated and treated wheat flour samples, according to CIE methods.

Samples		*L*—Lightness	*b*—Yellowness	*a*—Greenness	E*—Uniformity	Δ*E*—Color Difference
Untreated Flour	Control	79.7 ± 0.7 ^c^	17.4 ± 0.23 ^c^	0.73 ± 0.25 ^a^	81.6	0.0
Treated Flour	1 min	80.2 ± 0.8 ^c^	16.8 ± 0.20 ^b^	1.33 ± 0.08 ^b^	81.9	0.9
	3 min	84.3 ± 2.6 ^b^	17.1 ± 0.74 ^c^	1.21 ± 0.03 ^c^	86.1	4.6
	5 min	84.8 ± 1.0 ^a^	16.6 ± 0.28 ^a^	1.20 ± 0.51 ^d^	86.4	5.1

## Data Availability

Data is contained within the article or Supplementary Material.

## Supplementary Materials

The following supporting information can be downloaded at: https://www.mdpi.com/article/10.3390/foods12102056/s1.
